# The Manipulation of the Lipid Mediator Metabolism as Adjunct Host-Directed Therapy in Tuberculosis

**DOI:** 10.3389/fimmu.2021.623941

**Published:** 2021-03-12

**Authors:** Arista Nienaber, Frank E. A. Hayford, Ebrahim Variava, Neil Martinson, Linda Malan

**Affiliations:** ^1^Centre of Excellence for Nutrition, North-West University, Potchefstroom, South Africa; ^2^Department of Nutrition and Dietetics, School of Biomedical and Allied Health Sciences, College of Health Sciences, University of Ghana, Accra, Ghana; ^3^Perinatal HIV Research Unit, University of Witwatersrand, Soweto, South Africa; ^4^Department of Internal Medicine, Klerksdorp Tshepong Hospital Complex, North West Department of Health, Klerksdorp, South Africa

**Keywords:** cyclooxygenase, lipoxygenase, non-steroidal anti-inflammatory drugs, omega-3 polyunsaturated fatty acids, tuberculosis, lipid mediators, pharmaconutrition

## Abstract

Host-directed therapies (HDTs) enhance the host response to tuberculosis (TB) infection to reduce disease severity. For instance, the manipulation of lipid mediator production diminishes the hyperactive immune response which is a known pathological feature of TB that generates lung tissue damage. Non-steroidal anti-inflammatory drugs (NSAIDs) and omega-3 long-chain polyunsaturated fatty acids (n-3 LCPUFA) are examples of such HDTs. In this mini-review, we recapitulate the literature available on the effects of NSAIDs and n-3 LCPUFA in TB as well as the immunological pathways underpinning these effects. Many NSAIDs have a great deal of data describing their effects and safety and in many jurisdictions are inexpensive, and sold over the counter in neighborhood convenience stores and supermarkets. The potential benefits of NSAIDs in TB are well-documented in pre-clinical studies. The reduction of pro-inflammatory lipid mediator production by inhibiting cyclooxygenase (COX) pathways with NSAIDs has been found to improve lung histopathology, bacterial control, and survival. Additionally, n-3 LCPUFA and its novel bioactive metabolites produced by COX and lipoxygenase (LOX) have been identified as safe and effective pro-resolving and antibacterial pharmaconutrients. Nevertheless, heterogeneous results have been reported in pre-clinical TB studies. Recently, the importance of the correct timing of NSAIDs and n-3 LCPUFA administration in TB has also been highlighted. This mini-review will provide a better understanding of the potential contribution of these therapies toward reducing inflammatory lung damage and improving bactericidal activity, especially during later stages of TB infection. It further highlights that clinical trials are required to confirm benefit and safety in TB patients.

## Introduction

Tuberculosis (TB) remains one of the leading causes of death globally (1). Additionally, multi-drug resistant TB (MDR-TB) and extensively drug-resistant TB (XDR-TB) patients are burdened by long, costly treatments, with substantial adverse and drug interaction effects and poor cure rates (2, 3). To facilitate treatment, host-directed therapies (HDTs) have been under investigation to augment traditional anti-tubercle treatment regimes. HDTs attempt to modify the host's immune response to reduce tissue damage and indirectly aid bacterial killing, therefore, it should not select drug resistance (4–7). The main objectives of such treatments are to reduce treatment times, post-treatment lung pathology and TB relapse rates (8).

Inflammation is important in host defense, but TB elicits a hyperactive inflammatory response and is characterized by chronic non-resolving inflammation. This exacerbated inflammation results in lung tissue necrosis and cavitation, also facilitating TB transmission (9, 10). Lipid mediators (LMs) are hormone-like substances enzymatically produced from polyunsaturated fatty acids (PUFA) via cyclooxygenase (COX), lipoxygenase (LOX), and cytochrome P450 (CYP450) pathways. A balance between pro-inflammatory and inflammation resolving LM production is of utmost importance from the initiation of the immune response to the resolution of TB infection (11). The manipulation of LMs can be useful as part of immunomodulatory therapy in TB and work synergistically or additively with other standard treatments (12, 13). The use of non-steroidal anti-inflammatory drugs (NSAIDs) has been investigated in this regard (14, 15).

A recent meta-analysis of clinical trials showed that anti-inflammatory medication and pharmaconutrition therapy (vitamin D) may aid in inflammation resolution and improved disease progression outcomes (16). Additionally, the pharmaconutrient omega-3 long-chain PUFA (n-3 LCPUFA) also modify LM production and may be an emerging therapy to consider (17). In this mini-review, we aim to summarize the literature available on the effects of NSAIDs and n-3 LCPUFA in TB as well as the immunological pathways supporting these effects.

## Chronic Non-Resolving Inflammation in Tuberculosis

One of the key pathological features of TB is that immune cells are recruited to pulmonary spaces, leading to the development of lung granuloma and alterations in lung tissue (lesion formation) (18–20). Granuloma formation is not only intended to separate the TB-infected macrophages from surrounding healthy tissues, but also to keep them in close contact with T cells (21, 22). However, under the direction of the TB pathogen, a hyperactive and non-resolving host immune and inflammatory response are elicited which eventually facilitate lung tissue damage (9, 21). Cavity formation from liquefied granuloma is the most destructive form of TB (21). This results partly from the host's exacerbated inflammatory response, where higher concentrations of plasma IFN-γ, TNF-α, IL-17, and IL-1β have been associated with cavitary TB (9). Unfortunately, in 14–100% of patients, cavities, scarring (fibrosis), and pleural adhesions persist, contributing to persistent abnormal lung function even after TB cure and the resultant lower quality of life (23–27). Therefore, controlling the prolonged exacerbated inflammatory response may benefit clinical outcomes. There is also a close connection between cytokine and LM networks in TB which will be discussed in more detail in the following section.

## Lipid Mediators in Tuberculosis

**Figure 1:**
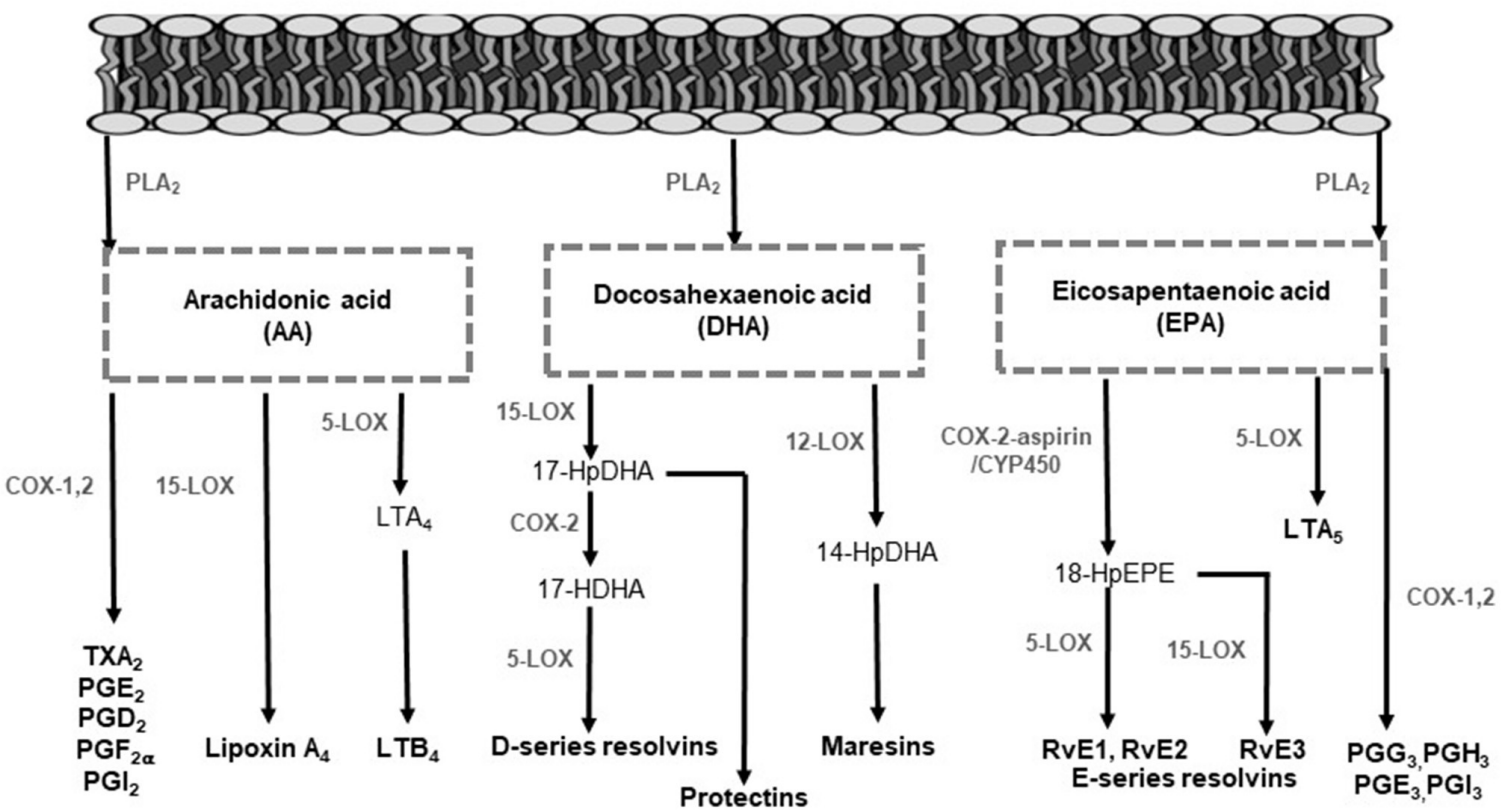
Lipid mediator biosynthesis pathways. In response to infection, polyunsaturated fatty acids are hydrolyzed from membrane phospholipids by phospholipase enzymes to release free fatty acids for lipid mediator production. Arachidonic acid serves as a substrate to form lipoxins, 4-series LTs, 2-series PGs, and TXs. The enzymes 5-LOX, 12-LOX, and 15-LOX produce LTs and lipoxins. Additionally, COX enzymes mediate the production of PGs and TXs. Eicosapentaenoic acid serves as a substrate for the intermediate 18-HPEPE by either COX-2 or CYP450 enzyme activity. From 18-HPEPE the E-series resolvins (RvE1, RvE2, and RvE3) are produced by 5- and 15-LOX. Eicosapentaenoic acid is also converted by 5-LOX to form the less inflammatory LTs. Docosahexaenoic acid is metabolized to form the D-series resolvins and protectins by 5- and 15-LOX and the maresins by 12-LOX. COX, cyclooxygenase; CYP450, cytochrome P450; HDHA, hydroxydocosahexaenoic acid; HpDHA, hydroxyperoxydocosahexaenoic acid; HEPE, hydroxyeicosapentaenoic acid; HpEPE, hydroxyperoxy-eicosapentaenoic acid; LOX, lipoxygenase; LT, leukotriene; PG, prostaglandin; PLA_2_, phospholipase A_2_; Rv, resolvins; TX, thromboxane.

PUFA are hydrolyzed from membrane phospholipids by phospholipase A_2_, to release free fatty acids locally at the site of infection or to be transported to the inflammatory site extracellularly (28–31). Released fatty acids give rise to LMs, by enzymatic pathways, to facilitate pro-inflammatory or inflammation-resolving responses (28, 31). In Figure 1, the LMs and their biosynthesis pathways are illustrated. Arachidonic acid (AA) is the main substrate for LM synthesis owing to its high concentrations in cell membranes (11). The LMs produced from AA include the lipoxins (LX), 4-series leukotrienes (LT), 2-series prostaglandins (PG), hydroxyeicosatetraenoic acids, and thromboxanes (TX) by CYP450, COX and LOX enzymes (Figure 1) (32, 33). The LMs derived from AA mostly signal pro-inflammatory responses, except for LX, which also display anti-inflammatory and pro-resolving effects (20, 34, 35). The n-3 LCPUFA eicosapentaenoic acid (EPA) and docosahexaenoic acid (DHA) also serve as precursors for LMs by COX and LOX activity. These LMs are referred to as specialized pro-resolving mediators (SPMs), including resolvins, protectins and maresins that promote anti-inflammatory pathways and actively contribute to inflammation resolution and tissue functioning restoration (36–40).

Recent studies have highlighted the impact of TB infection on systemic concentrations of different LMs, which persists even after anti-TB treatment (41–43). Apart from the important functions of LMs in the inflammatory response in TB, they also influence TB pathogenesis (41, 42). As such, LMs play a fundamental role in determining the fate of macrophages and their phagocytic ability, as well as immune cell recruitment (44, 45).

The functions of individual LMs in TB remain controversial but more recent research suggests that the balance and timing of the production of specific LMs during the TB disease course are essential for good treatment outcomes (12, 41, 44, 46). For example, the essential action of PGE_2_ in the innate immune response of human TB and how a balance in PGE_2_/LTB_4_ prevents severe inflammation and immunopathology (44, 47). Additionally, the AA-derived LXA_4_ has been positively correlated with inflammation and bacterial burden in TB patients (41). Furthermore, how PGE_2_, as well as other LM concentrations, affect outcomes may vary during the different stages of TB infection and, therefore, the specific roles of LMs may change during the disease progression (47–49).

Although research on the role of LM production and its manipulation as HDT in TB has focused mainly on AA-derived LMs, there is a growing interest in SPMs in TB. The plasma metabolomics of newly diagnosed human TB patients has revealed a pro-resolving plasma LM profile, including higher concentrations of the D-series resolvins (50). Furthermore, Colas et al. (12) reported that a pro-resolving LM profile (specifically resolvins) was correlated with 80-day survival, whilst lower levels of SPMs were linked to more severe disease in adults with TB meningitis (12). The reasons for this is that, apart from their inflammation resolving properties, maresins, resolvins and protectins have been implicated to enhance phagocytosis and anti-bacterial activity in TB (51). More studies are needed to describe the role of SPMs in TB and their immunotherapeutic properties. Nevertheless, the importance of LMs in TB regulation, together with the connection between cytokine and LM networks, accentuates the possibilities of LMs as immunotherapy targets in TB (52, 53). However, a time-dependant approach should be considered as the timing of the manipulation of these pathways may influence outcomes in TB disease (47, 48).

## Preclinical Trials on Cyclooxygenase- and Lipoxygenase-Modulating Drugs in Tuberculosis

The therapeutic effects of NSAIDs are mainly ascribed to their ability to reduce the production of pro-inflammatory LMs by inhibiting COX-1 and COX-2 activity (48, 54–56), but inadvertently the metabolism of pro-resolving LMs are also inhibited. In essence, they mitigate the conversion of AA to PGE_2_ and TXA_2_, thereby reducing pain, inflammation, fever, platelet aggregation and vasoconstriction (14, 48, 57). However, the major effects of NSAIDs in TB are ascribed to reduced PGE_2_ production, as PGE_2_ may inhibit phagocytosis while promoting bacterial growth and tissue damage in the late stages of TB-infection (47, 48). Aspirin (acetylsalicylic acid) and ibuprofen are frequently used NSAIDs (14). In murine models, low-dose aspirin (3 mg/kg/day) lower lung pathology and improve bacillary control thereby increasing survival (15, 58). This is ascribed to its anti-inflammatory effects at both systemic and local lung tissue level, together with lower neutrophil recruitment (by increased LXA_4_ and reduced LTB_4_ production) and enhanced T-helper1-(Th1) cell responses (15, 39, 58, 59). Although aspirin has been implicated in enhancing the antibacterial activity of pyrazinamide, it may display an antagonistic effect on isoniazid (55, 60). On the other hand, it seems that ibuprofen may be a better anti-inflammatory agent option, displaying no interference with anti-tuberculosis therapy in rodent models (60). Furthermore, when provided in the absence of conventional TB treatment, improvements in lung histopathology, survival and bacillary load have been reported when administering ibuprofen (80 mg/kg/day) in TB-infected mice (57).

Other NSAIDs displaying COX-inhibiting characteristics include indomethacin and diclofenac. In an earlier study, Hernandez-Pando et al. (61) found that when administering 5 mg/kg/day indomethacin to BALB/C mice with TB-induced lung granulomas, the T cell imbalances, that are characteristic of TB infection, were reversed and the harmful cell-mediated and humoral immunity lessened (61). In an *in vitro* study in blood samples of TB patients, COX-2 was found to be upregulated. However, the COX-1/2 inhibitor indomethacin reduced cytokine responses and T cell proliferation by modulating Th1 effector and T regulatory cells (62). Additionally, indomethacin enhanced the response to immunization with *M. vaccae* (63). Similarly, diclofenac treatment has been shown to reduce lung lesions and bacillary load and increase survival in murine models (64, 65). The new generation NSAID celecoxib also selectively inhibits COX-2 but has fewer side effects (44). It can increase the sensitivity of bacteria to antibacterial treatment and reverse MDR-TB (66, 67). This is ascribed to COX-2 regulating the MDR protein 1 (MDR-1) gene expression. Therefore, the administration of celecoxib blocks the MDR efflux pump and increases drug accumulation (66).

In preclinical TB studies, COX inhibition by NSAID therapy has also had some unfavorable effects. Both ibuprofen and celecoxib treatment increased bacterial burden and ibuprofen decreased survival in *Mtb*-infected mouse models (68). The detrimental properties of NSAID therapy could be attributed to its effects on the adaptive immunity impairing Th1 cell responses and mitigating IFN-γ expression (68). However, it seems that the infection route may influence outcomes as earlier preclinical studies showing promising results infected mice intravenously causing acutely high systemic bacterial loads and inflammation (68). Furthermore, the timing of NSAID administration is important. When administering ibuprofen to *Mtb*-infected mice on day one following infection, lung pathology and inflammation were increased which was linked to PGE_2_ inhibition early in the onset of the disease. Conversely, inhibition later in the disease (60 days after infection) reduced neutrophil inflow and, thereby, lessened lung pathology (69). Therefore, COX inhibition may be detrimental to host resistance early in TB infection (48, 70).

With regards to modulating LOX pathways, inhibiting 5-LOX reduces lung pathology, whilst improving bactericidal activity and survival rates (44, 49, 71). Furthermore, 5-LOX deficient mice also show increased IFN-γ, IL-12 and nitric oxide synthase mRNA levels since LX negatively regulates Th1 cell responses (71). When 5-LOX deficient mice were treated with LTB_4_ susceptibility toward TB, lung inflammation and tissue damage were worsened, demonstrating the key role of LTB_4_ on TB progression and disease outcomes (44). Various LOX-inhibiting therapies exist, such as selective redox-based inhibitors, iron ligand inhibitors e.g., zileuton, and thiazoles e.g., Zeneca ZD2138, but whether they can be successfully repurposed as HDT in TB is to be determined.

## Clinical Trials Investigating the Use of Cyclooxygenase-Inhibiting Drugs in Tuberculosis

There are several limitations when translating animal research findings to humans, therefore, the success of the use of COX-inhibiting therapy in preclinical trials prompted the initiation of clinical trials. Observational research has caused concern that NSAID use may increase the risk of the development of active TB. In case reports and an unadjusted analysis of a case-control study, NSAID treatment positively associated with an increased risk of active TB (72–75). However, it is unclear whether this association was causal or rather related to the fact that individuals with subclinical, diagnosed, or undiagnosed active TB are known to have increased NSAID use (75). Furthermore, in the case-control study, COX-inhibition was not associated with active TB in an adjusted analysis. The results were also not replicated in rheumatoid arthritis patients where NSAID therapy was not associated with the risk of active TB (75, 76). Supporting this, in a phase 1 *ex vivo* study in healthy human whole blood inoculated with *Mtb*, celecoxib did not affect whole-blood bactericidal activity (77). Therefore, these findings should be interpreted carefully and more controlled trials are required.

There is a paucity in randomized clinical trials exploring the use of NSAIDs as an adjunct treatment during active TB. In older studies, low-dose aspirin reduced some of the side effects of pyrazinamide treatment in TB patients (78, 79). Aspirin has also been investigated as adjunctive treatment in TB meningitis patients, where different dosages of aspirin daily (81, 150, or 1,000 mg) ensued fewer strokes and lower 3-month mortality rates (80, 81). The beneficial effect was ascribed to aspirin inhibiting TXA_2_ and increasing protectin concentrations in cerebral spinal fluid (81). In 2019 a randomized controlled phase 2 trial of the efficacy and safety of using adjunctive ibuprofen in XDR-TB (NCT02781909) was completed, however, the results of this trial remain to be published. Two other trials are registered in this regard including a phase 1 trial administering etoricoxib to MDR-TB patients (NCT02503939) and a randomized controlled clinical trial administering meloxicam to TB patients to determine its ability to modulate or prevent TB-immune reconstitution inflammatory syndrome (IRIS) (NCT02060006). A third three site EDCTP-funded trial is about to start recruiting randomizing drug sensitive and drug resistant TB patients to ibuprofen, aspirin or placebo. NSAIDs have well-known side effects (48). However, as is the case for most other anti-inflammatory drugs, no serious adverse effects related to NSAIDs have been reported in clinical trials in TB patients (14, 16). Compared to traditional antibiotic treatment, NSAIDs are not subject to bacterial resistance and some may even aid in improving bacterial sensitivity to antibiotics (44). Nevertheless, the newer generation NSAIDs may be a safer option to consider. Furthermore, the results of the clinical trials that are pending, will provide greater clarity on the safety and efficacy of NSAID therapy in TB (44). Prospective randomized clinical trials should focus on the dosage, timing and duration that provide the best results when administering NSAIDs adjunct to TB treatment.

## Fatty Acid Manipulation as Pharmaconutrition Therapy in Tuberculosis

Apart from the possibility of using drug therapy to modulate COX and LOX activity, a therapeutic nutritional approach to alter the substrate for COX and LOX pathways may be a promising way to get the same results with fewer side effects. This could be possible through the use of n-3 LCPUFA as pharmaconutrition therapy. Previous studies on the role of n-3 LCPUFA in TB are limited. Some of these studies have raised awareness that supplementation may cause an increased active TB susceptibility and reduced ability of the host to control the infection (82–85). Bonilla et al. (83) found that *fat-1* mice with a genetically higher n-3 PUFA status were more susceptible to active TB and that bacterial loads positively associated with n-3 PUFA levels. The authors ascribed this to the macrophages of these mice which were deficient in various important functions (83). Supporting this, n-3 LCPUFA-fed *Mtb*-infected guinea pigs had a higher bacterial burden when compared with their n-6 PUFA-fed counterparts (84, 85). In addition to these studies, Bazinet et al. (86) found that n-3 PUFA supplementation in piglets, increased the levels of antibodies in response to TB immunization (86).

Contrasting with these results, n-3 LCPUFA supplementation has been shown to lower bacterial load, compared with n-6 PUFA-supplemented or control groups in *Mtb*-infected mice (87). Recently, it was also found that EPA and DHA supplementation initiated 1 week after *Mtb* infection induced a more pro-resolving lung LM profile, and exerted both local lung and systemic anti-inflammatory effects, whilst enhancing bactericidal activity and improving anemia of infection in C3HeB/FeJ mice (17). The reason for inconsistent findings may be related to the timing of the administration of n-3 LCPUFA. When administered after the initial inflammatory response to the infection, beneficial effects were found, whilst providing it before or early in *Mtb* infection worsened the outcomes. Differences in EPA and DHA dosages may also have contributed, where a higher EPA content seems beneficial (17, 87). Lastly, due to the preclinical nature of the studies, the type and species of animals used may have influenced results (83–85). The safety and efficacy of n-3 LCPUFA as therapy adjunct to standard TB treatment and how this compares to other anti-inflammatory treatments are still to be determined in preclinical trials. However, preliminary results from a TB mouse model study conducted by our group show that n-3 LCPUFA does not interfere with the efficacy of standard TB medication (unpublished data).

Only two clinical trials have been conducted to ascertain the effect of n-3 LCPUFA in TB. The first supplemented a combination of fish oil (350 mg n-3 PUFA), vitamin A (1,500 UI) and Zinc (10 mg) with standard TB drug treatment, in pediatric TB patients. The group receiving supplementation for 1 month had lower TNF-α concentrations and an improved body mass index, compared with a group that received standard drug treatment only (88). In the second trial, n-3 LCPUFA (300 mg) was supplemented in combination with Zinc sulfate (15 mg) once per day for 1 month in a small number (*n* = 20) of adult Indonesian TB patients receiving standard TB treatment (89). Supplementation caused non-significant, reduced sputum smear conversion rates and mediated significant improvements in body weight and CD4^+^ counts compared with the control group (89). However, in both studies, the timing of the initiation of supplementation was not mentioned and n-3 LCPUFA was supplemented in combination with other nutrients. Although there is a paucity in clinical trials on n-3 LCPUFA supplementation in TB, it has been found safe in animal TB models and clinical trials in other inflammatory diseases (17, 32). As pharmaconutrition therapy, n-3 LCPUFA supplementation is also safe for long term use and not subject to bacterial resistance like antibiotics. Bearing in mind the side effects of other anti-inflammatory drugs the nutritional modulation of inflammatory pathways may be a safer approach. However, as clinical evidence is lacking, future randomized clinical trials should provide n-3 LCPUFA as single pharmaconutrient adjunct to standard TB treatment. Furthermore, the appropriate timing, duration and dosage of such supplementation need to be investigated as the manipulation of LM concentrations may produce different outcomes depending on the stage of TB disease.

## Discussion

**Figure 2:**
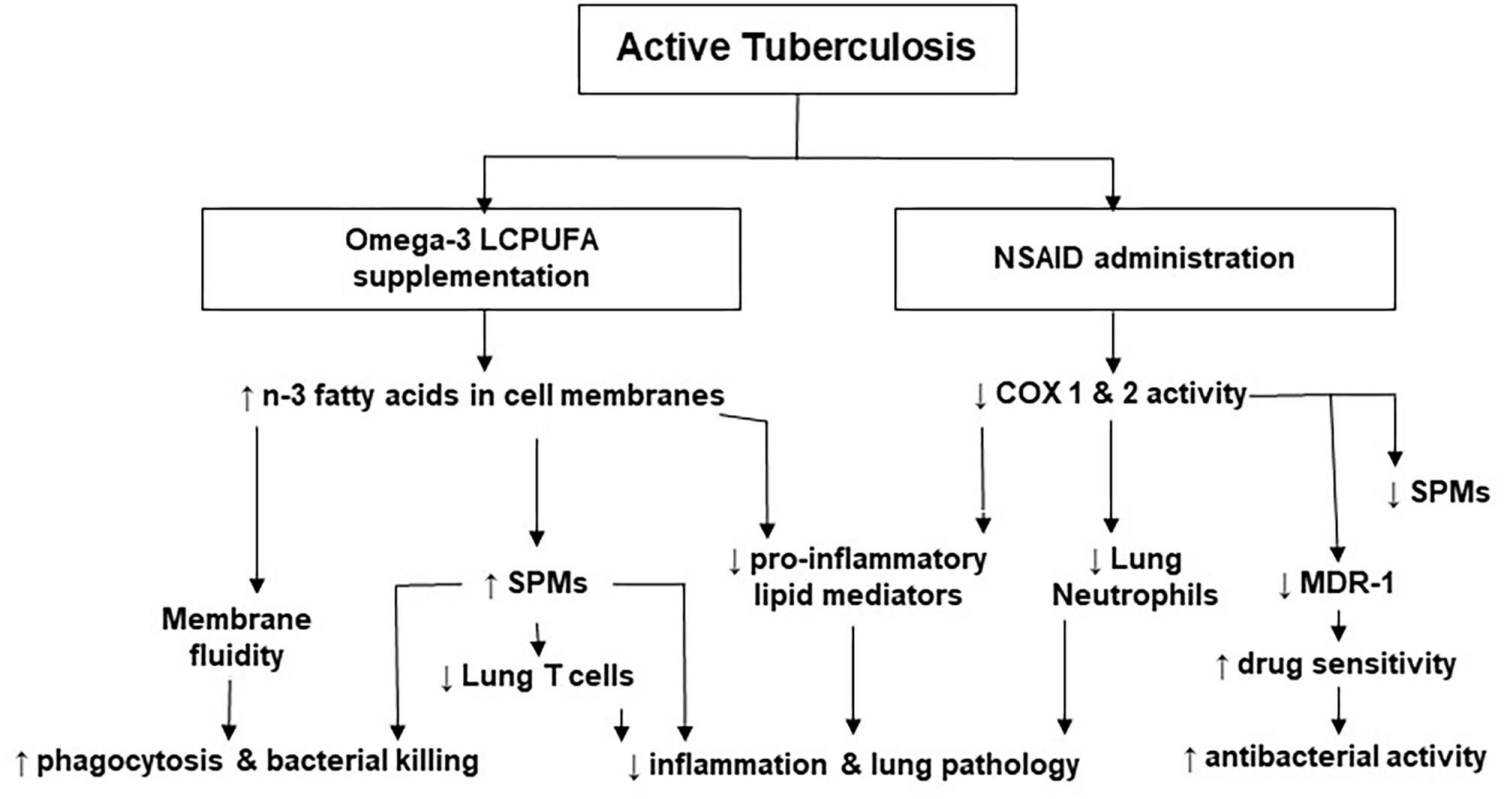
The effects of non-steroidal anti-inflammatory drugs and omega-3 long-chain polyunsaturated fatty acids in tuberculosis. COX, cyclooxygenase; LCPUFA, long-chain polyunsaturated fatty acid; MDR, multi-drug resistant; NSAID, non-steroidal anti-inflammatory drug; n-3, omega-3; SPMs, specialized pro-resolving lipid mediators.

Published data suggest that LMs regulate inflammatory and immune responses and that their roles vary at different stages of the disease. For example, high concentrations of PGE_2_ may worsen disease progression and down-regulate cell-mediated immunity in later stages of TB infection (69). Altering LM concentrations by modulating COX and LOX activity is a novel HDT approach in TB. Figure 2 represents the effects of NSAIDs and n-3 LCPUFA in TB as well as the underlying mechanisms supporting them. Prescribing NSAIDs as analgesic and anti-inflammatory medication is common worldwide. These medications have shown promising results in pre-clinical TB studies by inhibiting COX activity to reduce the production of pro-inflammatory and sometimes immunosuppressive LMs (14, 15, 60, 65, 90). This aids in attenuating inflammation-induced tissue damage and improves the antibacterial actions of the host with active TB. Additionally, COX-inhibitors can aid in improving the concentrations of certain drugs and drug sensitivity, by the manipulation of MDR-1 (66, 77). Therefore, the synergistic effects of TB treatment and NSAIDs may benefit TB outcomes (14, 55, 62, 91). Preclinical trials on NSAID therapy in TB have also highlighted that the timing of administration is important, where NSAIDs at later stages of the disease may be more beneficial (48, 68, 69). Although favorable results regarding the anti-inflammatory and antibacterial activity of COX-inhibition therapy in TB have been found in preclinical trials, more randomized controlled clinical trials are needed to determine the efficacy and safety in patients with active TB (14, 15, 55, 57, 58, 60, 92). More definite recommendations are anticipated upon completion and publication of clinical trials that are currently ongoing.

Apart from beneficial effects, NSAIDs also carry well-known side effects, such as the risk of gastrointestinal ulcers, bleeding and renal injury (48). Another HDT option is n-3 LCPUFA which facilitate pro-resolving and anti-inflammatory pathways by altering the membrane phospholipid fatty acid composition of blood and tissue cells that are important in immune responses (93–95). These fatty acids partially replace AA in membranes as the substrate for pro-inflammatory LMs (94, 96, 97). Furthermore, they also serve as precursors for SPMs, which have inflammation resolving properties (36–40). They alter immune cell recruitment by halting neutrophil infiltration and lowering T cell proliferation (31, 32, 98). Also, SPMs have direct effects to stimulate monocytes to migrate and differentiate into macrophages for phagocytic activity, and to enhance bacterial phagocytosis and killing (99–102). The few available studies on n-3 LCPUFA in TB have portrayed mixed results with some showing benefit concerning bacterial killing and pulmonary inflammation (17, 83, 87), whilst others reported harm (82–85, 103). A recent study in *Mtb-*infected mice highlighted the importance of the timing of n-3 LCPUFA supplementation, where supplementation after the initial inflammatory response seems to be beneficial (17). Preclinical studies combining n-3 LCPUFA with standard TB drug treatment are still required. In the only two clinical trials that have been conducted on n-3 LCPUFA therapy in TB patients, a positive effect was found on sputum smear conversion, body weight gain, inflammation resolution, and CD4^+^ T cell count (88, 89). As n-3 LCPUFA were combined with other nutrients in these clinical trials, more randomized controlled trials are required to determine the correct dosage and timing of supplementation in patients with active TB. Another possible HDT in TB is LOX-manipulating therapy, however, clinical trials on repurposing drugs such as zileuton are still lacking.

## Conclusion

Both NSAIDs and n-3 LCPUFA may help to reduce excessive inflammatory lung damage and improve bactericidal activity, especially during later stages of TB disease. However, more human data, particularly randomized controlled clinical trials are required to confirm the clinical benefit and safety of these HDT approaches in patients with active TB.

## Author Contributions

LM and AN contributed to the conception of the mini-review. AN was responsible for text writing and figure assembly of the first draft. AN, LM, FH, EV, and NM revised and edited the text and figures. All authors contributed to the article and approved the submitted version.

## Conflict of Interest

The authors declare that the research was conducted in the absence of any commercial or financial relationships that could be construed as a potential conflict of interest.
